# *BrCYP71A15* Negatively Regulates Hg Stress Tolerance by Modulating Cell Wall Biosynthesis in Yeast

**DOI:** 10.3390/plants12040723

**Published:** 2023-02-06

**Authors:** Ali Anwar, Shu Zhang, Lixia Wang, Lilong He, Jianwei Gao

**Affiliations:** Institute of Vegetables, Shandong Academy of Agricultural Sciences, Jinan 250100, China

**Keywords:** Chinese cabbage, Hg stress, *BrCYP71A15*, GFP, yeast, cell wall, gene expression

## Abstract

Over the past two decades, heavy metal pollution has been a common problem worldwide, greatly threatening crop production. As one of the metal pollutants, Mercury (Hg) causes damage to plant cells and reduces cellular and biochemical activities. In this study, we identified a novel cytochrome P450 family gene, *BrCYP71A15*, which was involved in Hg stress response in yeast. In Chinese cabbage, the *BrCYP71A15* gene was located on chromosome A01, which was highly expressed in roots. Additionally, the expression level of *BrCYP71A15* was induced by different heavy metal stresses, and the *BrCYP71A15* protein exhibited a strong interaction with other proteins. Overexpression of *BrCYP71A15* in yeast cells showed no response to a number of heavy metal stresses (Cu, Al, Co, Cd) in yeast but showed high sensitivity to Hg stress; the cells grew slower than those carrying the empty vector (EV). Moreover, upon Hg stress, the growth of the *BrCYP71A15*-overexpressing cells increased over time, and Hg accumulation in yeast cells was enhanced by two-fold compared with the control. Additionally, *BrCYP71A15* was translocated into the nucleus under Hg stress. The expression level of cell wall biosynthesis genes was significantly influenced by Hg stress in the *BrCYP71A15*-overexpressing cells. These findings suggested that *BrCYP71A15* might participate in HG stress tolerance. Our results provide a fundamental basis for further genome editing research and a novel approach to decrease Hg accumulation in vegetable crops and reduce environmental risks to human health through the food chain.

## 1. Introduction

Chinese cabbage belongs to the Cruciferae family that originated in China [1], which has become one of the essential economic vegetable crops and is widely cultivated around the globe [2,3]. Over the past two decades, heavy metals have been considered a severe environmental threat to all living organisms, such as plants, animals and humans [4]. Global population has shown an intensive increment due to industrialization, during which excessive amounts of heavy metal pollutions such as mercury (Hg), cadmium (Cd), chromium (Cr), aluminum (Al), arsenic (As) and lead (Pb) have been produced [5,6]. Among these heavy metals, Hg is highly toxic even under low concentrations [6]. In plants, there are no specific channels to absorb and transport Hg from the root to the shoot [4,7]. The excessive accumulation of Hg causes physiological and biochemical disorders and decreases plant productivity [8,9,10]. Moreover, the uptake of Hg by the plant can be transferred to the human body, leading to numerous chronic disorders such as skin cancer, heart problems and lung diseases [10,11].

Hg accumulation is influenced by soil Hg contamination in plants, which is absorbed through the root system and translocated to the shoot [12]. The enhanced accumulation of Hg increases ROS production; reduces the activities of antioxidant enzymes; decreases the production of nutrients, hormones and pigments; and negatively affects photosynthetic capacity [11,12]. A previous study reported that Hg accumulation in plants might stimulate the production of ROS, which leads to damage to proteins and membrane lipids [11]. The overproduction of ROS can influence the activities of essential enzymes such as GSH, SOD, POD, CAT, APX and GR [12]. The GR enzyme is highly sensitive to Hg stress and significantly reduced in alfalfa roots [4,11,13,14]. Hg stress damages root activities, interrupts water and nutrient absorption and reduces uptake capacity from the root to the shoot [11]. Hg stress also shows a great effect on chlorophyll contents, photosynthesis and plant production [6]. Therefore, it is crucial to investigate Hg stress and identify candidate genes that are potentially involved in Hg stress tolerance.

Plant cytochrome (CYP) 450s are the most prominent enzyme family, which is widely involved in NADPH/O_2_-defendant hydroxylation reactions in plants [14]. In addition, the CYP genes play a fundamental role in the biosynthesis of secondary metabolites, phytohormones and antioxidants in plants [15]. CYPs have been reported to play an essential role in plant hormone biosynthesis and signaling pathways, while these hormones are potentially involved in plant abiotic stress responses, such as high and low temperature, drought, salinity and heavy metal stress [14,16]. Up to now, millions of CYP genes have been identified in different species, and about 16,000 genes have been reported in plant species [14]. A number of CYP genes have shown significant responses to abiotic stresses, exhibiting great potential to overcome plant resilience to environmental influences [16,17]. For example, the *ABA8Ox* (*CYP707A*) gene is upregulated under drought stress in maize [18]. The *CYP707A1* and *CYP707A2* genes are highly expressed under osmotic stress and drought stress in *Arabidopsis* and *Arachis hypogaea*, respectively [19]. *CYP96A8* is involved in lignin biosynthesis and drought stress responses [14,18]. In *Arabidopsis*, the *CYP86A2* mutant reduces cuticle membrane thickness and enhances water permeability in response to drought stress [20]. In sorghum, *CYP71A25* and *CYP71B2* are upregulated under drought stress [14,15,16]. The CYP genes play a significant role in maintaining ROS hemostasis to enhance abiotic stress tolerance. *TaCYP81D5* and two *Arabidopsis* genes (*AtCYP709B3* and *AtCYP81D8*) enhance salinity stress tolerance by facilitating the ROS scavenging activities in wheat and *Arabidopsis*, respectively [14,15,17,21]. The *CYP88A* gene exhibits an enhanced expression level under Al toxicity in wheat [14,22]. The *CYP81D8* gene, putatively involved in metabolism, shows a four-fold increase in the expression in *Arabidopsis* under AL stress.

In this study, we identified a CYP gene, *BrCYP71A15,* in Chinese cabbage, which is involved in Hg stress tolerance. In Chinese cabbage treated with Hg stress, the expression level of the *BrCYP71A15* gene was enhanced. The *BrCYP71A15* gene was cloned to yeast using the pRS416 vector to investigate its functions in response to Hg stress, which exhibited high sensitivity to Hg stress. The pRS416-GFP vector was used to test the subcellular localization of the *BrCYP71A15* gene under salinity stress in yeast. Our study will be helpful for the understanding of the function of the *BrCYP71A15* gene and genetic modification of Chinese cabbage, which can improve crop production and adaptation to environmental cues. It will be more interesting to explore the role of the *BrCYP71A15* gene in the hormone signaling pathway and ROS scavenging to identify novel mechanisms in Chinese cabbage. Future research on *BrCYP71A15* will offer the possibility of genetically engineered crop varieties with enhanced crop production.

## 2. Results

### 2.1. The Effect of Hg Stress on the Chlorophyll Content

**Figure 1:**
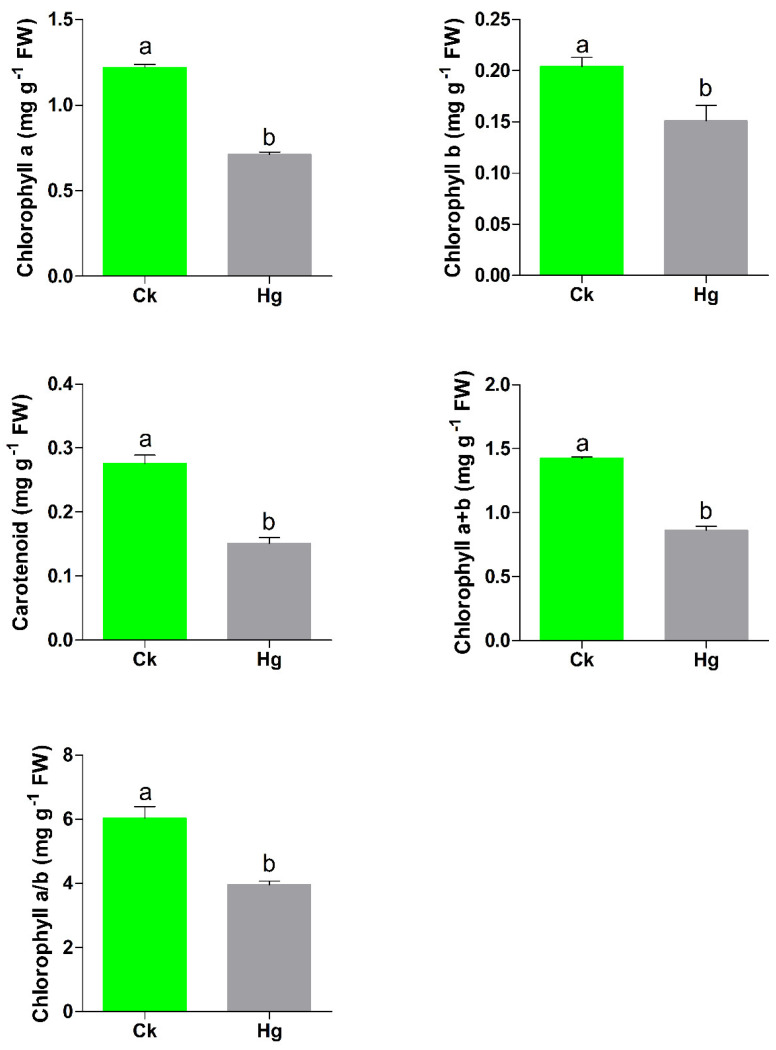
The effects of Hg stress on the chlorophyll content in Chinese cabbage seedlings. Different letters above the bar indicate significant difference at *p* < 0.05.

The chlorophyll content is extremely sensitive to environmental influences and degrades very quickly. In this study, Chinese cabbage seedlings were exposed to Hg for 7 days, and the chlorophyll content was determined. The results indicated that Hg stress had a detrimental effect on chlorophyll. The chlorophyll a content was reduced by 41.72% under Hg stress compared with that of CK. Similarly, chlorophyll b, carotenoid, chlorophyll A+B and chlorophyll A/B contents were reduced by 26.10%, 45.25%, 39.48% and 34.32%, respectively, compared with those of CK as presented in Figure 1. These findings indicate that Hg stress significantly reduced the chlorophyll content, thereby greatly hindering plant growth and production.

### 2.2. BrCYP71A15 Gene Induced by Hg Stress in Chinese Cabbage

**Figure 2:**
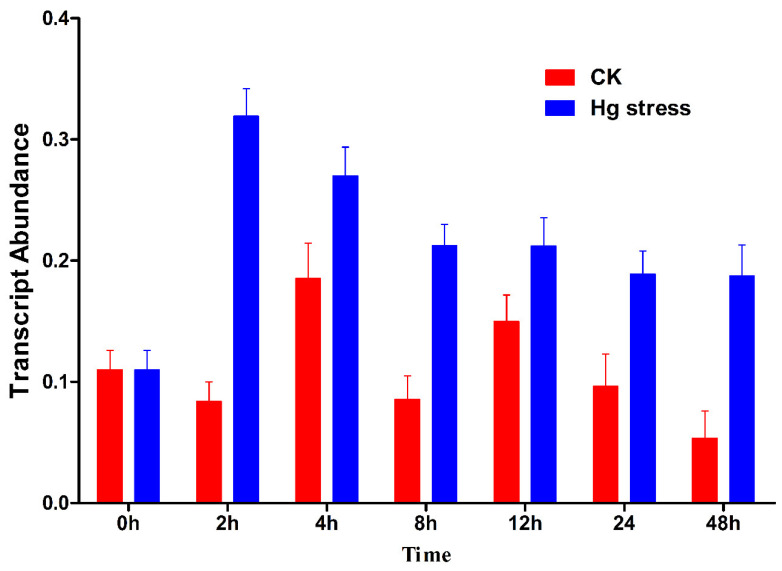
*BrCYP71A15* transcription abundance under Hg stresses in Chinese cabbage. The transcript abundance was determined by qRT-PCR in Chinese cabbage seedlings treated with 100 mM Hg. Error bars indicate ± standard deviation.

To explore the transcript abundance of the *BrCYP71A15* gene under Hg stress, Chinese cabbage seedlings were exposed to 100 μM Hg stress. The whole plant samples were collected at different times, i.e., 0, 2, 4, 8, 12, 24 and 36 h after Hg stress. The quantitative reverse transcription PCR (RT-qPCR) results showed that the mRNA abundance was the same at 0 h but three-fold higher than that of CK and then gradually decreased over time as presented in Figure 2. The transcript abundance between CK and samples under Hg stresses was significantly different. These results indicate that the *BrCYP71A15* gene might play an important role in response to Hg stress.

### 2.3. Expression Patterns of the BrCYP71A15 Gene under Different Abiotic Stresses

**Figure 3:**
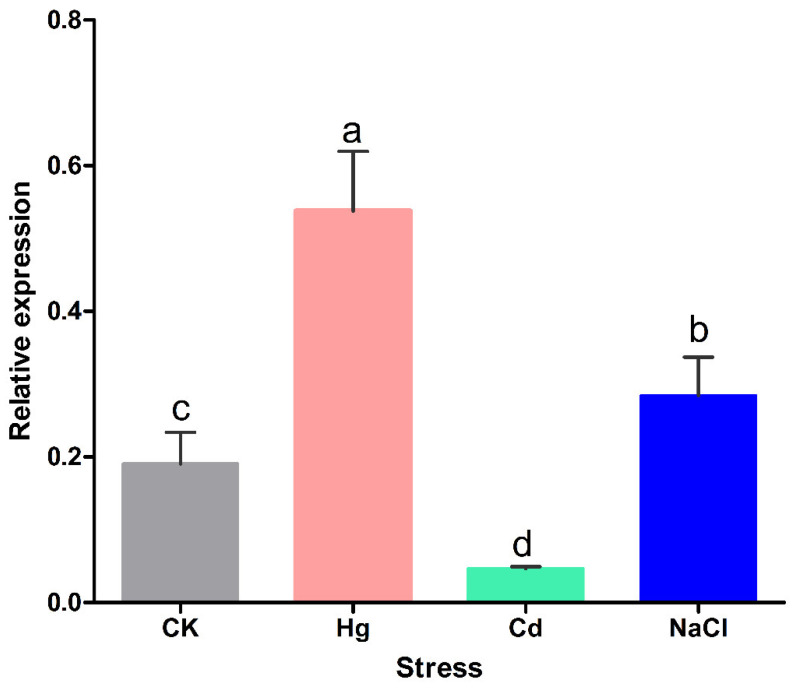
The expression level of the *BrCYP71A15* gene under NaCl, Cd and Hg stresses. Colors indicate different stresses, and letters above the error bar represent significant differences at *p* > 0.005.

The *BrCYP71A15* gene plays an important role in regulating plant response to abiotic stresses. However, its function is yet to be fully understood. To confirm the molecular mechanism of the *BrCYP71A15* gene under abiotic stresses, their expression level was investigated under Hg, Cd and NaCl stresses and compared with the CK (Control), as presented in Figure 3. The results indicate that the *BrCYP71A15* gene was highly expressed under Hg stress, followed by NaCl stress. The expression level of *BrCYP71A15* was reduced under Cd stress compared with that of CK and those under Hg and NaCl stresses (Figure 3). These findings suggested that the transcript level of *BrCYP71A15* was significantly induced by stress and *BrCYP71A15* might be involved in Hg stress tolerance in Chinese cabbage.

### 2.4. Expression Patterns of the BrCYP71A15 Gene in Different Tissues of Chinese Cabbage

**Figure 4:**
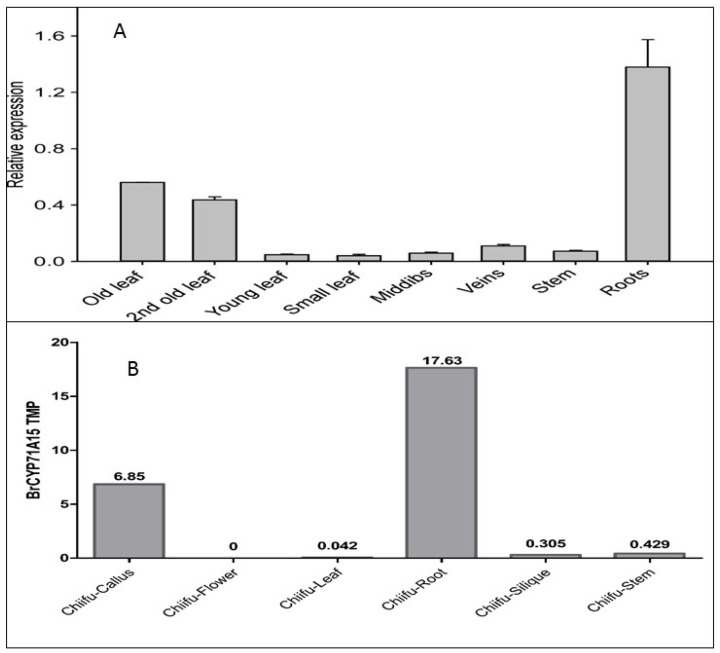
The expression of *BrCYP71A15* in different tissues of Chinese cabbage (**A**), and TPM data (**B**) were download from Chinese cabbage database (http://brassicadb.cn/#/, accessed on 18 August 2022).

To investigate the role of the *BrCYP71A15* gene in plant growth and development, we explored its expression in different tissues of Chinese cabbage. The results suggested that the *BrCYP71A15* gene was highly expressed in roots, followed by older leaves, as presented in Figure 4A. However, the expression level of the *BrCYP71A15* gene was reduced in young leaves, midribs, leaf veins and stems, compared with that of roots and old leaves. The TPM (transcript per million) value was obtained from a Chinese cabbage database (http://brassicadb.cn/#/, accessed on 18 August 2022). These findings suggest that the *BrCYP71A15* gene was highly expressed in root tissues (17.63), followed by callus (6.85). The expression of the *BrCYP71A15* gene in leaf, silique and stem was the lowest compared with that of the root and callus (Figure 4B). The *BrCYP71A15* gene showed no expression in flower tissues. Taken together, these findings indicate that the *BrCYP71A15* gene was actively expressed in Chinese cabbage, specifically in roots, which could play vital roles in growth and developmental processes of Chinese cabbage. Thus, it is important to discover its functions on the *BrCYP71A15* gene under Hg stress tolerance.

### 2.5. BrCYP71A15 Protein–Protein Association Network

**Figure 5:**
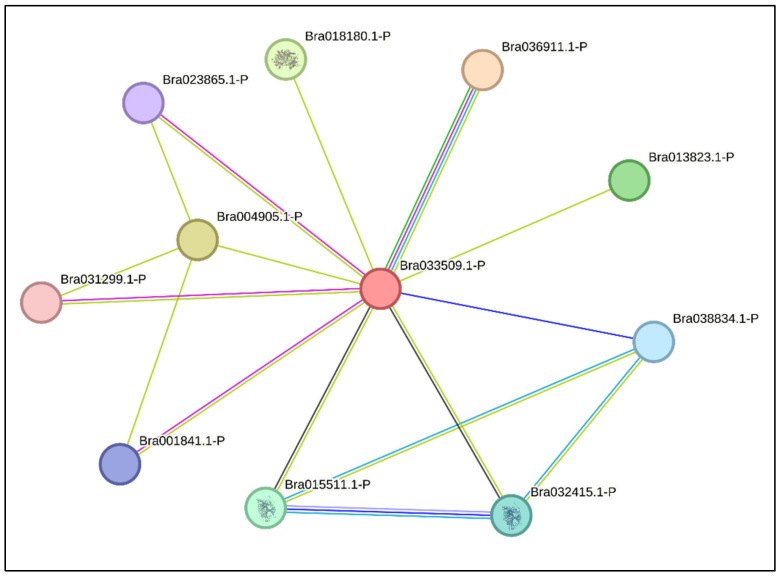
*BrCYP71A15* protein–protein association network.

The STRING database was used to investigate the protein–protein network of *BrCYP71A15*. The PPA network contained 10 proteins, 11 nodes and 16 edges, with a local clustering coefficient of 0.876 and a PPI enrichment *p*-value of 0.079 (Figure 5). Different values were assigned to each node. The edges were assigned with different scores, with darker edges representing higher scores, indicating it had stronger interactions with other proteins. The results showed that a number of proteins, including *Bra036911* (Ferredoxin c 1), *Bra004905* (uncharacterized protein), *Bra018180* (Alpha-glucan phosphate 2), *Bra013823* (uncharacterized protein), *Bra015511* (4-hydroxyphenylpyruvate dioxygenase), *Bra032415* (4-hydroxyphenylpyruvate dioxygenase), *Bra038834* (uncharacterized protein), *Bra038834* (uncharacterized protein), *Bra023865* (uncharacterized protein) and *Bra031299* (uncharacterized protein), exhibited higher degrees of connections. The connection between *Bra036911* and *BrCYP71A15* protein was significant, indicating they might directly interact with each other.

### 2.6. BrCYP71A15 Overexpression in Response to Abiotic Stresses in Yeast

**Figure 6:**
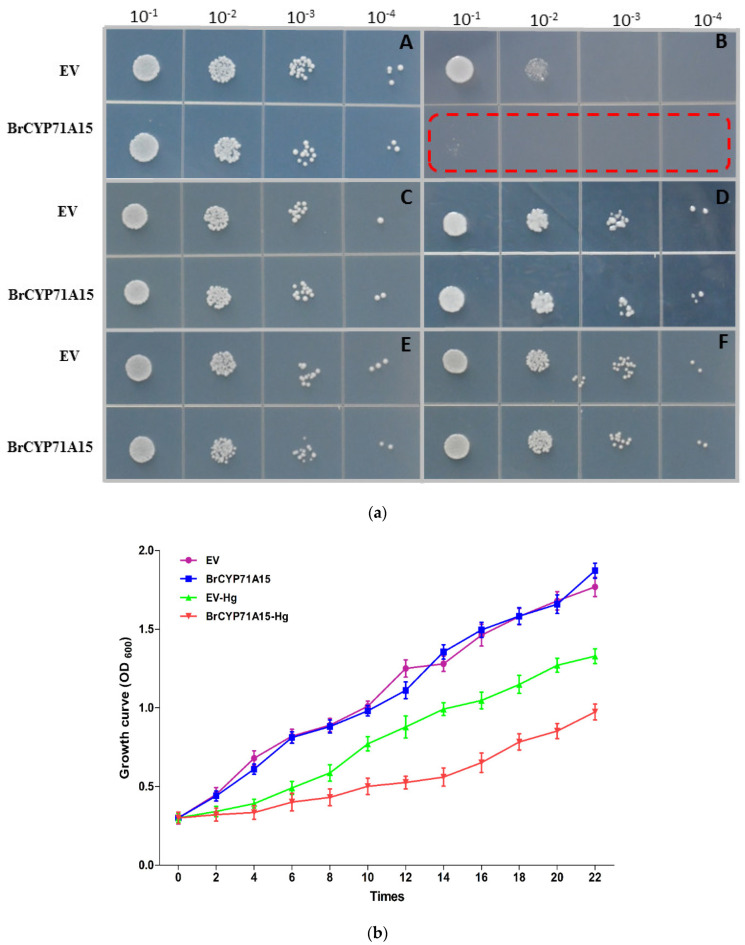
(**a**) The *BrCYP71A15* response to heavy metal stress tolerance in yeast. Empty vector (yeast wild-type) and *BrCYP71A15* OE cells were grown in URA liquid medium for 24 h at 30 °C. OD600 was adjusted to 0.3, and the cells were subjected to different types of abiotic stresses, including URA (Control) (A), Hg (B), Cd (C), Al (D), Cu (E) and NaCl (F). The upper mentioned folds represent the serial 10-fold dilution (the starting concentration (OD_600_) is 0.3. All experiments are repeated three times. (**b**) The growth curves of *BrCYP71A15*-overexpressing cells under 100 mM Hg stress. Yeast cells with EV (empty vector (Yeast WT)) and expressing *BrCYP71A15* were grown at 30 °C. Cell density was monitored at 0, 2, 4, 6, 8, 10, 12, 14, 16, 18, 20, 22 and 24 h after the treatment. Error bar represents the deviation of three independent replications.

To elucidate the function of the *BrCYP71A15* gene in abiotic stress tolerance, we generated a *BrCYP71A15* overexpressing yeast model using the pRS416-GFP vector. The *BrCYP71A15* overexpressing cells were exposed to abiotic stresses (75 μM Cd, 100 mM Hg, 100 μM Al, 50 μM Cu and 1 M NaCl), as presented in Figure 6. The results show that the cells exhibited no response to Cd, Al, NaCl and Cu stresses. However, the expression of *BrCYP71A15* was highly sensitive to Hg stress compared with EV (Figure 6b), and thus, *BrCYP71A15* might play a significant role in response to Hg stress.

**Figure 7:**
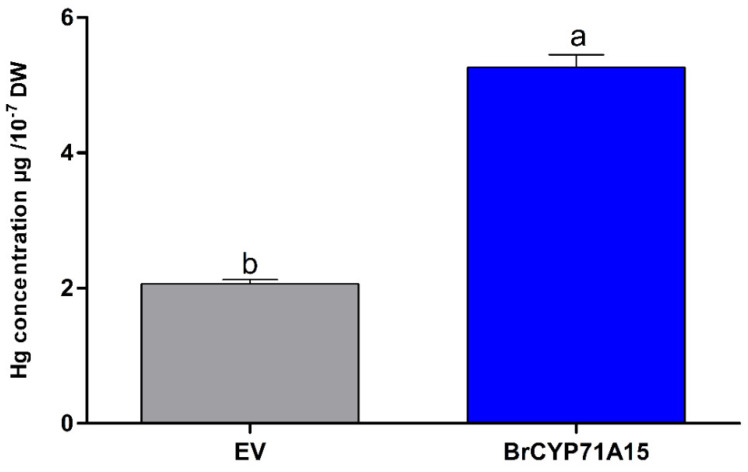
Hg concentration in yeast cells. Yeast strains expressing EV and *BrCYP71A15* were grown at 30 °C for 2 d. The cells were collected in liquid SC with 100 mM Hg and the OD600 values were measured using an atomic absorption spectrometer. Error bars indicate ± SD of three independent experiments. Letters above the error bar represent significant differences at *p* > 0.005.

Furthermore, we conducted the growth curve of the yeast cells overexpressing *BrCYP71A15* without and with Hg stress, as presented in Figure 7. Compared with EV, the transgenic cells show more sensitivity than those EV-expressing cells. Under Hg stress, there was no significant difference between EV and *BrCYP71A15*-overexpressing cells at 6 h. Then, the growth of the EV-expressing cells gradually became faster, and a significant difference was observed compared with that of the *BrCYP71A15*-overexpressing cells. These results indicate *BrCYP71A15* played a key role in Hg stress tolerance.

### 2.7. Subcellular Localization of the BrCYP71A15 Gene

**Figure 8:**
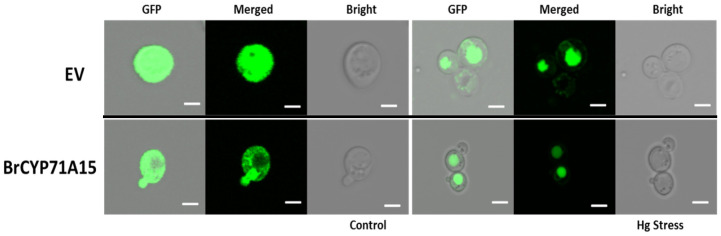
The subcellular localization of *BrCYP71A15* and empty vector tagged with GFP and transiently expressed in yeast cells treated with 100 mM Hg. The images were obtained from GFP, merged and bright channels. Scale bar: 10 μm.

There is no evidence supporting the translocation of the *BrCYP71A15* gene into the nucleus under Hg stress. To confirm the subcellular localization, the *BrCYP71A15* gene was transiently expressed in fusion of GFP in yeast and the fluorescence was observed through a confocal microscopy. Under control conditions, the *BrCYP71A15* gene existed in the nucleus as dot-like structures, as presented in Figure 8. When treated with Hg stress, the *BrCYP71A15* protein was translocated into the nucleus. These findings suggested that the *BrCYP71A15* gene could specifically enter the nucleus under Hg stress.

### 2.8. Cell Wall Biosynthesis Gene

**Figure 9:**
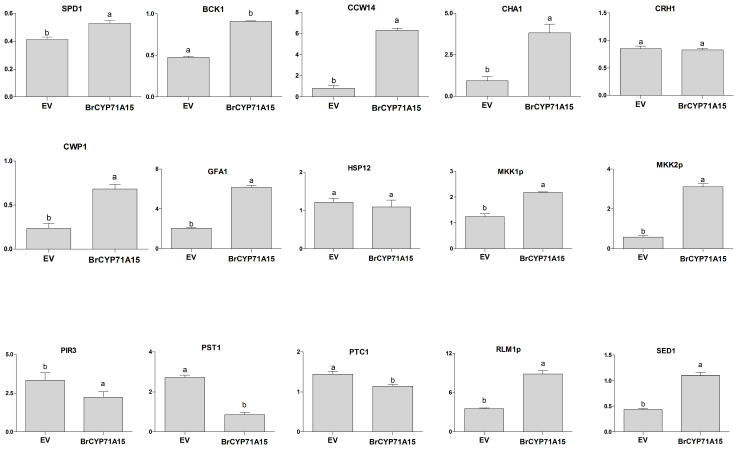
qRT-PCR analysis was used to assess the expression level of cell wall biosynthesis genes under Hg stress in yeast. Error bar represents the deviation of three independent replications, and letters above error bar represents significant differences at *p* > 0.005.

The cell wall is considered as a key barrier for heavy metals, triggering rescue mechanisms to maintain cellular integrity, and yeast is a convenient system to study the function of the cell wall against heavy metal [23,24] . In this study, we investigate the expression levels of genes that are involved in cell wall biosynthesis, remodeling, metabolism and signal transduction under different abiotic stresses. The results suggested that *SPD1*, *BCK1*, *CCW14*, *CHA1*, *CWP1*, *GFA1*, *MKK1p*, *MKK2p*, *RIM1p* and *SED1* genes were expressed in *BrCYP71A15*-overexpressing yeast cells under Hg stress compare with the control (Figure 9). However, *PST1*, *PIR3* and *PTC1* genes were significantly downregulated in *BrCYP71A15*-overexpressing yeast cells under Hg stress. The expression of *HSP12* and *CRH1* genes showed no difference between the EV and *BrCYP71A15*-overexpressing cells, as presented in Figure 9. Cell wall biosynthesis-related genes showed a dynamic response to Hg stress, which might be involved in *BrCYP71A15* gene activation under Hg stress.

## 3. Discussion

Plants under heavy metal stresses can enhance ROS production, which is highly toxic in nature and causes detrimental effects on chlorophyll, protein, hormonal biosynthesis and enzyme activities [25,26]. Plants have evolved a series of pathways to cope with ROS production to minimize the harmful effects of heavy metal stress, including antioxidant enzymes, hormones, transcription factors and stress responsive genes [12]. Among various heavy metals, mercury (Hg) is considered the most toxic heavy metal, exhibiting strong harmful effects on plant growth and development [5]. Hg stress disrupts many molecular, biochemical, physiological, cellular mechanisms, thereby greatly reducing plant growth and productivity [6]. In the present study, we reported that Hg stress resulted in a significant reduction in the chlorophyll content of plants (Figure 1). A previous study reported that Hg stress increases ROS accumulation and reduces the chlorophyll content [27], which is consistent with our findings. Plants exposed to heavy metal stresses show increased heavy metal accumulation, which leads to the overproduction of ROS such as H_2_O_2_ and O_2_ [13,25]. Numerous studies have reported that heavy metal stresses can cause deleterious effects on plant growth and decreased chlorophyll contents due to the overproduction of ROS [5,6]. These findings suggested that Hg stress might increase the overproduction of ROS and lead to a significant reduction in chlorophyll accumulations in Chinese cabbage.

The *BrCYP71A15* gene has high similarity with the *Arabidopsis* gene CYP71A15 (AT5G24950). *BrCYP71A15* is a constituent family member of putative cytochrome P450, which controls plant growth, seed development, germination and other physiochemical and biochemical characteristics [15]. The putative cytochrome P450 is ubiquitously founded in plant species [14]. Recent studies show that CYP714 family genes are involved in gibberellin deactivation and homeostasis through 16a, 17-epoxidation or 13-hydroxylation in rice and *Arabidopsis* [14]. The protein–protein association network suggests that the dynamic interaction and correlation of *BrCYP71A15* with other proteins (Figure 5) might be involved in regulation and activation under stress conditions. However, the functions of the *BrCYP71A15* gene have not been directly reported. Here, we provide evidence which shows that *BrCYP71A15* might regulate Hg stress tolerance in yeast.

A number of genes are involved in hormonal control of plant growth, development and biotic and abiotic stress responses. This work demonstrates that *BrCYP71A15* is potentially involved in Hg stress response. As presented in Figure 4, *BrCYP71A15* genes show different transcription levels (TPM) in various tissues and are highly expressed in root tissues. The expression pattern of genes is a significant indication for biological functions and molecular mechanisms [7]. Plants uptake and translocate Hg from soil through roots to different tissues, and we report that *BrCYP71A15* was mostly expressed in roots, which might be the reason that the plant had high sensitivity to Hg stress (Figure 6). Root is the key organ that has early contacts with Hg and is also responsible for the absorption and translocation of Hg, and thus, it might be the reason that *BrCYP71A15* has high expression in roots (Figure 1). A previous study reported Cyp96b4/dss1 mutants regulate ABA biosynthesis and accumulation to enhance drought stress tolerance in rice [17]. Similarly, the *CYP709B3* gene plays a key role in the regulation of salinity stress tolerance and enhances the expression level in *Arabidopsis* [16]. Based on these results, it can be concluded that the expression level of *BrCYP71A15* was increased (Figure 2 and Figure 3) and hence the plant exhibited enhanced sensitivity to Hg stress (Figure 6).

miRNAs are a class of noncoding RNA molecules with a length of 20–24 bp, which play a key role in plant growth and abiotic stress tolerance [25]. These miRNAs are considered as a central regulator of transcription activation under stress conditions [5]. We reported 10 miRNAs targeting the *BrCYP71A15* gene, which might be involved in various developmental processes including abiotic stress responses (Table 1). In *Brassica,* miR160 shows a significant response to Cd stress, and its transcript level is elevated, while miR164b and miR394s are upregulated under sulfate deficiency [5,28,29]. Cd stress increases the expression of MiR156, MiR171, MiR393 and MiR396a in roots of *B. naps* [5]. Likewise, *Medicage truncatula* has been treated with Hg, Al and Cd to investigate the expression level of miRNAs. miR171, miR319, miR393 and miR529 are activated when exposed to heavy metal stresses (Hg, Al and Cd) [5,30]. These findings suggest that miRNAs are potentially involved in heavy metal stress tolerance. In this study, we reported that *BrCYP71A15* regulated the expression of miRNAs that might be involved in Hg stress tolerance response (Table 1).

The *BrCYP71A15*-overexpressing cells showed high sensitivity to Hg stress (Figure 6a,b), and the accumulation of the Hg content was elevated (Figure 7). A previous study reported that the cell wall plays a significant role in the protection of cells from abiotic stresses [31]. The cell wall provides a strong barrier to environmental influences and reduces harmful effects [31,32,33]. In yeast, the transcriptional reprogramming can activate the transcript level of genes involved in cell wall biosynthesis, signal transduction and stress responses [31,33]. Studies have reported that SLT2, MAPK and MAPKK1 are involved in cell wall biosynthesis [31,34]. In this study, Hg stress activated the transcript levels of cell wall biosynthesis gene in *BrCYP71A15* (Figure 9). The expression of SPD1, BCK1, CCW14, CHA1, CWP1, GFA1, MKK1p, MKK2p, RIM1p and SED1 genes was elevated when exposed to Hg stress, but the expression of PST1, PIR3 and PTC1 was downregulated. These results indicate that *BrCYP71A15* interacts with cell wall biosynthesis genes when exposed to Hg stress. Protein Kinase C (PKC1) is regulated by BCK1p, MKK1p, SLT2p and ROM2p, which plays a fundamental role in cell wall biosynthesis, maintenance and cell integrity [23,31]. These findings are supported by a previous study, which reported that S1Fa OE cells regulate cell wall biosynthesis genes under salt stress in yeast [4]. Hence, it can be concluded that the *BrCYP71A15* gene interacts with cell wall biosynthesis genes, and thus, Hg sensitivity is enhanced, and the accumulation of the Hg content is increased in yeast cells. Interestingly, under Hg stress, *BrCYP71A15* was located in the nucleus (Figure 8), which might promote the transcription of cell wall biosynthesis genes. Taken together, the *BrCYP71A15* gene was highly expressed in roots and showed high sensitivity to Hg stress. Thus, the Hg content might be increased in yeast cells, and the expression of cell wall biosynthesis genes might be activated. These findings are in line with previous studies, which reported that salt stress reduces the antioxidant enzymatic activities, enhances the ROS and MDA contents in S1fa cells and regulates the transcript level of genes involved in cell wall integrity [4]. In summary, it can be concluded that the *BrCYP71A15* gene exhibits high sensitivity to Hg stress and increases Hg accumulation, which may play a key role in cell wall biosynthesis under Hg stress. Further investigations are needed to identify downstream genes to fully understand the function of the *BrCYP71A15* gene in response to Hg stress.

## 4. Materials and Methods

Chinese cabbage (Cv. Guangdongzao) seeds were presoaked with 1% sodium hypochlorite for 3 min and then washed at least five times with ddH_2_O to remove excessive sodium hypochlorite from the seed surface. The seeds were germinated in ½MS media in a controlled growth chamber, transferred to a hydroponic culture and incubated for five more days before being treated with 50 μM Cd, 100 mM Hg and 1 M NaCl, respectively. The samples were collected after two days after treatments and ground in liquid nitrogen to extract the total RNA [4].

### 4.1. Chlorophyll Contents

The total chlorophyll contents were determined by extraction in 95% ethanol and measured using a spectrophotometer. As described previously, the absorbance levels were measured at 470, 649 and 666 nm [35].

### 4.2. Yeast Constructs

The yeast (Saccharomyces cerevisiae) cells (GRY472 cells) were used in this study (https://www.yeastgenome.org/, accessed on 18 August 2022) to validate the function of gene [8]. To construct the yeast (*Saccharomyces cerevisiae*) overexpression vectors, the coding sequence of Chinese cabbage *BrCYP71A15* gene was cloned into the pRS416-GFP vector. The coding sequence of the *BrCYP71A15* gene was amplified from Chinese cabbage cDNA with specific primers (Supplementary Table S1) and then inserted into the SPE1 site on pRS416-GFP using the infusion cloning kit (TAKARA Catalog no. 011614; Clontech) [4]. The sequence insertions were confirmed through SANGER sequencing and then used to investigate abiotic stress tolerance in yeast. To determine the subcellular localization of the *BrCYP71A15* protein, the *BrCYP71A15* gene was inserted into pRS416-GFP. The subcellular localization of the fusion protein was observed under a Ziess Axiophot fluorescence microscope, as described previously [4].

To predict the miRNAs, the coding sequence of the *BrCYP71A15* gene was submitted to the psRNATarget server (https://www.zhaolab.org/psRNATarget/, accessed on 18 August 2022) [4].

### 4.3. Hg Concentration in Yeast Cells

Yeast strains grown on SC solid plates with 100 µM HgCl_2_ at 30 °C for 2 d. Cells were collected in liquid SC, and the OD_600_ was recorded before atomic absorption spectrometer measuring. Hg^2+^ content was measured with the 7700X ICP-MS (Agilent) [36].

### 4.4. Tolerance Assay and Growth Curve

The final pRS416-GFP vector-overexpressing yeast cells cultured in the URA medium were diluted until the OD_600_ value was 0.1 and then were incubated again until the OD_600_ reached 0.3. The cell culture was then diluted four-fold and treated with 75 μM Cd, 100 mM Hg, 100 μM Al, 50 μM Cu, 1 M NaCl and URA (control), respectively, and incubated at 30 °C for five days [9]. No treatment was added for the control. The photos were taken after five days of incubation, and the experiment was repeated three times. The *BrCYP71A15* overexpressing yeast cells without and with the Hg treatment were grown at 30 °C in a liquid URA culture medium and diluted until the OD_600_ value was about 0.1. The cells were incubated again until the OD_600_ value reached 0.3, and then the OD_600_ was recorded every 2 h to prepare the growth curve of the cells [9].

### 4.5. Total RNA Extraction and qRT-PCR Analysis

The total RNA was extracted for Chinese cabbage tissues using TRIzol, while yeast RNA was extracted using the M5 EASYspin yeast RNA rapid extraction kit, MF158-01 (Mei5 Biotechnology, Co., Ltd.). For yeast RNA extraction, the cells were grown until the OD_600_ value reached 0.3 at 28 °C and then treated with Hg for 18 h before the total RNA was harvested [4]. The first-stand cDNA was synthesized using a PrimeScript and RT reagent kit with gDNA Eraser (TAKARA). The SYBR Premix Ex-Taq Kit (TAKARA) was used for quantitative real-time PCR. All experiments were performed with three independent biological replications. The transcript levels were calculated using the 2^ΔΔ-^CT method. The TMP values of Chinese cabbage tissues were obtained from the Chinese cabbage database (http://brassicadb.cn/#/, accessed on 18 August 2022) for *BrCYP71A15* gene. The primers used for qRT-PCR are presented in Supplementary Table S1.

### 4.6. Statistical Analysis

Three independent biological replications were used for each treatment, and the whole experiment was repeated three times. The data were statistically analyzed using an analysis of variance and compared with the control using the LSD test (*p* > 0.05) by using the Statistix 8.1 software (https://www.statistix.com/, accessed on 18 August 2022). The Graphpad Prism 5 software was used for graphical presentation [4].

## 5. Summary

In this study, we identified *BrCYP71A15*, which participates in the immediate response to Hg stress in yeast cells. The *BrCYP71A15* gene is expressed in different tissues of Chinese cabbage, while mostly expressed in root tissues. The expression level of the *BrCYP71A15* gene increased over time when exposed to Hg stress. Compared with other abiotic stresses, the expression level of the *BrCYP71A15* gene was the highest under Hg stress. The overexpression of *BrCYP71A15* showed high sensitivity to Hg stress, and the accumulation of Hg was increased in yeast cells, which, in turn, regulated the expression of cell wall biosynthesis genes. Thus, it can be concluded that the *BrCYP71A15* gene is potentially involved in Hg stress response. Further comprehensive studies are required to explore the physiological and molecular mechanisms of *BrCYP71A15* in Hg stress tolerance, which will improve protected vegetable crop production as well as provide a strong background for genetic crop implements.

## Figures and Tables

**Table 1:** Prediction of miRNAs targeting the *BrCYP71A15* gene of Chinese cabbage.

miRNA	Gene	Length	Start	End	miRNA Aligned Fragment	Alignment	Targeted Fragment	Inhibition
ath-miR5644	Bra033509	20	81	100	GUGGGUUGCGGAUAACGGUA	::: :: :::.::::.::	CACCUCUAACCGUAACCUAC	Cleavage
ath-miR8183	Bra033509	21	232	252	UUUAGUUGACGGAAUUGUGGC	....:.::.:::::.::.:	CUUGUAGUUUCGUCAGCUGAC	Cleavage
ath-miR854a	Bra033509	21	217	237	GAUGAGGAUAGGGAGGAGGAG	.: : ::::::::::..:	GGUCGCGUCCCUAUCCUUGUA	Cleavage
ath-miR854b	Bra033509	21	217	237	GAUGAGGAUAGGGAGGAGGAG	.: : ::::::::::..:	GGUCGCGUCCCUAUCCUUGUA	Cleavage
ath-miR854c	Bra033509	21	217	237	GAUGAGGAUAGGGAGGAGGAG	.: : ::::::::::..:	GGUCGCGUCCCUAUCCUUGUA	Cleavage
ath-miR854d	Bra033509	21	217	237	GAUGAGGAUAGGGAGGAGGAG	.: : ::::::::::..:	GGUCGCGUCCCUAUCCUUGUA	Cleavage
ath-miR854e	Bra033509	21	217	237	GAUGAGGAUAGGGAGGAGGAG	.: : ::::::::::..:	GGUCGCGUCCCUAUCCUUGUA	Cleavage
ath-miR2934-5p	Bra033509	21	733	753	UCUUUCUGCAAACGCCUUGGA	:..: :: :::: .::::.::	UUUAUGGAGUUUCUAGAAGGA	Cleavage
ath-miR426	Bra033509	21	1268	1288	UUUUGGAAAUUUGUCCUUACG	: :::::::. ::.:::.	UUUCAGGACAAGAUUUCAAGU	Cleavage
ath-miR5632-3p	Bra033509	21	808	828	UUGGAUUUAUAGUUGGAUAAG	::: : ..:: ::::.:::	GAUAUGCUGUUACAAAUUCAA	Cleavage

## Data Availability

Not applicable.

## Supplementary Materials

The following supporting information can be downloaded at: https://www.mdpi.com/article/10.3390/plants12040723/s1, Table S1: Primer used in this study.
