# Supplementary material for: BrCYP71A15 Negatively Regulates Hg Stress Tolerance by Modulating Cell Wall Biosynthesis in Yeast
Source: Plants (Basel). 2023 Feb 6;12(4):723. doi: 10.3390/plants12040723 (PMC9966778; doi:10.3390/plants12040723)
Supplement: Supplementary file 1 [file plants-12-00723-s001.zip › plants-2185739-supplementary.pdf]

Table S1. Primer used in this study.

**Primer Name**	**Yeast qRT-PCR Primer**
Mkk1p-RT-F	CCCAGAACCACTGACCTCTT
Mkk1p-RT-R	GGTACGAGTTCGGGTAGTGT
Mkk2p-RT-F	GTTGAAACGTGGCGGTAGAA
Mkk2p-RT-R	CACTGACACCGAAATCGCAT
RLM1-RT-F	GCTCAAAGACAAACCCAGCA
RLM1-RT-R	CTTCGATGGCAGTGAACCAG
SDP1-RT-F	CGAACATCGCACCAAAGAGT
SDP1-RT-R	GCACGAATTCACGGTCTTCA
Ptc1-RT-F	GGGAGCTTCCGGATTCAGTT
Ptc1-RT-R	TATCAGGCCACCTGCTTGTT
Bck1-RT-F	TTAGAAGTCGTCGCAGCCAT
Bck1-RT-R	AAGAAGCTCGTTAGCGGTTG
Cha1-RT-F	GCTGCAACAGCATGTCAAAG
Cha1-RT-R	GGGCTCAATGACCTGAGAGT
Hsp12-RT-F	AAGGTCGCTGGTAAGGTTCA
Hsp12-RT-R	TGGACACGACCGGAAACATA
SED1-RT-F	CCACTTTCACCACAAACGGT
SED1-RT-R	GGTTGATGTGGTTGTTGGCT
PST1-RT-F	CACCATTGGCCAAACCTTCA
PST1-RT-R	AGGTTGTCGAGACCACCAAT
CRH1-RT-F	ATTGAATGGGTGGGTGGTGA
CRH1-RT-R	TCCATCGAGGTACCAAGTCG
Ccw14-RT-F	AGACTCCATCTGCCCAAACA
Ccw14-RT-R	GGAATCGCCCAATGAAGCAT
GFA1-RT-F	AGCGTCTGACTTTCTGGACA
GFA1-RT-R	GGTATCCGCAGTTTCACCAC
PIR3-RT-F	ACCCGTGGTCCACTTTAACT
PIR3-RT-R	CAGTAGTAGTGGCAGCCTGT
CWP1-RT-F	GTGGCTCATTTGAGGCAACT
CWP1-RT-R	ACCCGTCCTTGATAGCGTAG
Yeast Actin-RT-F	ATATTCCAGGATCAGGTCTTCCGTAGC
Yeast Actin-RT-R	GTAGTCTTCTCATTCTGTTGATGTTGTTGTTG

Cloning primers

Primer Name	Primers
BrCYP71A15-F	ATGGAAATGATATCAATCTCTCTGTGCT
BrCYP71A15-R	CTAAAGAGTAGATGAAGCCAAAGATGGG
BrCYP71A15-F	tagaactagtggatcATGTCTATTTTCATTTGTTTTCTCTTACTCTTCC
BrCYP71A15-R	gcagcccgggggatcTCACCAGCGATGAAGATTTGGTAC
